# Biology and Ecology of *Delia planipalpis* (Stein) (Diptera: Anthomyiidae), an Emerging Pest of Broccoli in Mexico

**DOI:** 10.3390/insects14070659

**Published:** 2023-07-24

**Authors:** Guadalupe Córdova-García, Laura Navarro-de-la-Fuente, Diana Pérez-Staples, Trevor Williams, Rodrigo Lasa

**Affiliations:** 1 Red de Manejo Biorracional de Plagas y Vectores, Instituto de Ecología AC (INECOL), Xalapa 91073, Mexico; azolla29@gmail.com (G.C.-G.); mlnavarrodelafuente@yahoo.com.ar (L.N.-d.-l.-F.); 2 INBIOTECA, Universidad Veracruzana, Av. de las Culturas Veracruzanas 101, Xalapa 91090, Mexico; diperez@uv.mx

**Keywords:** lifecycle, longevity, sexual behavior, sexual receptivity, oviposition behavior, genetic identity

## Abstract

**Simple Summary:**

The development of effective pest management tools for the radish fly, *Delia planipalpis*, requires an understanding of the basic biology and reproductive behavior of this pest. In the present study, we investigated the pest’s biology on radish and confirmed the identity of the pest by sequencing the *COI* gene. The development of immature stages lasted 32–33 days. Female flies began mating 1–4 days after emergence, whereas males attempted to mate immediately after emergence. Most females only mated once during the 4-day observation period. Females preferred to lay eggs on radish plants rather than broccoli, although this result may have been influenced by the plant used to rear the insect colony. Male and female flies lived longer when individualized (14.5–22.5 days) compared to insects that were confined in groups (10–11 days). This is the first detailed and statistically rigorous study on the basic biology of this important pest of broccoli and other cruciferous crops.

**Abstract:**

*Delia planipalpis* (Stein) (Diptera: Anthomyiidae) is a pest of crucifers, such as broccoli, radish, cauliflower, turnip and cabbage. It has been recently described in Mexico as a significant emerging pest of broccoli. Due the lack of knowledge of this pest, the present study aimed to determine its life cycle, female sexual maturation, copulation, oviposition behavior and adult longevity. The identity of the fly in Mexico was confirmed genetically by sequencing the cytochrome oxidase subunit 1 gene (*COI*). The mean development time of *D. planipalpis* was 32–33 days on radish at 24 °C under laboratory conditions. Females became sexually mature 1–2 days after emergence, and the highest incidence of matings was recorded on the second day (60%). Under choice conditions, *D. planipalpis* females preferred to oviposit on radish plants, rather than broccoli plants, possibly due to the use of radish for rearing the laboratory colony. Oviposition and the mean number of eggs laid varied among the broccoli varieties, with the highest oviposition observed on the Tlaloc variety. Repeated attempts to rear the laboratory colony on broccoli plants failed. Radish-reared insects of both sexes lived longer when individualized in the adult stage (14.5–22.5 days) than when adult flies were maintained in groups (10–11 days). This study contributes to the understanding of *D. planipalpis* biology and provides information that can be used to establish future control strategies against this pest.

## 1. Introduction

Several flies belonging to the genus *Delia* (Diptera: Anthomyiidae) are pests of a range of field crops in temperate zones [1,2,3]. The cabbage root fly, *Delia radicum* L., the onion fly, *Delia antiqua* (Meigen), and the bean seed fly, *Delia platura* (Meigen), are the species of *Delia* that have been studied in the greatest detail [3,4,5]. *Delia* is a taxonomically difficult group within the Anthomyiinae subfamily [6]. Recently, *Delia planipalpis* (Meigen), (formerly *Hylemya planipalpis* (Stein)) also known as the radish fly, was reported to be infesting broccoli plants in the states of Mexico (near Mexico City), Guanajuato and Puebla in Mexico [7]. Species identification was performed based on the morphological characteristics of the larvae and pupae collected from infested broccoli in Guanajuato in 2020 [8]. Both of these studies revealed that about 80–100% of the specimens collected from damaged broccoli plants were *D. planipalpis,* and a small percentage corresponded to *D. platura*.

The literature on *D. planipalpis* is scarce although the first reference to its presence in North America dates back to 1915 in California [9]. Subsequent reports of damage by this pest originate from the Canadian prairies [1] and the western provinces of North America, from the Yukon south to Mexico [10]. It is a polyphagous pest whose larvae mainly feed on the radish’s root (*Raphanus sativus* L.) [5,10], but can also attack other wild and cultivated plants belonging to the family Brassicaceae. Among cultivated cruciferous plants, broccoli (*Brassica oleracea* L. var. italica), cauliflower (*B. oleracea* var. botrytis), cabbage (*B. oleracea* var. capitata), turnip (*Brassica rapa* L.) and canola (*Brassica napus* L.) are preferred [5,7]. In Mexico, this species has been reported as an emerging invasive pest in broccoli and other cruciferous plants causing economic losses that have increased considerably in the last decade [7,11]. The only study on the life history and ecology of *D. planipalpis* was that of Kelleher [10] in Manitoba, Canada, but the study was descriptive and based on small sample sizes (sometimes less than 10 flies). Since this date, no additional studies on the biology or ecology of this pest have been published.

An understanding of the biology and ecology of *D. planipalpis* is essential for the development of effective control strategies. The objective of this study was, therefore, to determine the oviposition behavior, immature development and adult longevity of the pest. We then examined the fly’s mating behavior and the effect of the host plant on sexual development under laboratory conditions. Finally, we compared the flies’ selection of host plant by offering radish or different varieties of broccoli plants. Given the taxonomic complexity of the genus and the recent appearance of the pest in central Mexico, we began by performing a genetic confirmation of the identity of the pest.

## 2. Materials and Methods

### 2.1. Laboratory Colony

A colony of *D. planipalpis* was started at the Instituto de Ecología AC, Xalapa, Veracruz, Mexico, using adults that emerged from pupae infesting broccoli plants in San José Iturbide, Guanajuato (21°9′23″ N; 100°18′50″ W, 2142 m elevation) in January 2020. Pupae were placed in groups of 15–20 in transparent plastic cups (200 mL) containing vermiculite moistened with 0.3% (*w*/*v*) sodium benzoate solution (Sigma-Aldrich, Mexico City, Mexico). Cups were kept in acrylic mesh-covered cages (60 × 60 × 90 cm) for adult emergence and maintained in a climatically controlled room at 24 ± 1 °C, 65 ± 10% relative humidity (RH) and 12:12 h (L:D) photoperiod. Males and females were kept together for 4–8 days in cages before several radish plants (taproot and leaves) were exposed inside the cage to allow female oviposition. Radish plants were previously cleaned using 0.1% hypochlorite solution, washed with tap water, and the lateral leaves were cut 1 cm from the stem leaving only two of the central leaves. Infested radishes were recovered after 7 days and placed in vermiculite trays for larval development and pupation. Flies of both sexes for use in experiments were collected from adult emergence cages at an age of 1–4 days and were assumed to be sexually mature and mated. Prior to laboratory experiments, all adult flies had continuous ad libitum access to water and to a 3:1 mixture of sugar and hydrolyzed yeast. All the experimental procedures described in the following sections were performed in a climate-controlled laboratory at 24 ± 1 °C, 65 ± 10% relative humidity (RH) and 12:12 h (L:D) photoperiod.

### 2.2. Morphological and Genetic Identification of the Pest

For taxonomic and genetic confirmation of the pest’s identity, different samples of larvae, pupae and adults were collected in 2022 from the established colony and from damaged broccoli plants and traps in a field in Guanajuato, Mexico. Taxonomic identification was performed using the keys of Savage et al. [6] and Meraz-Álvarez et al. [7] and involved identifying specific characters of larvae, pupa and adults, including the male genitalia. Genetic confirmation was performed using two adults from the colony (one male, one female), two adults (one male, one female) collected from yellow sticky traps and two larvae collected from damaged broccoli inflorescences in Guanajuato. Field samples were collected from the El Colgado and Cuatro Esquinas broccoli farms near the town of Dolores Hidalgo (21°17′29″ N; 100°54′15″ W, 1920 m elevation). Two adult flies (one male and one female) of *D. platura* were also collected from a yellow sticky trap in El Colgado for taxonomic and genetic identification. Genomic DNA was extracted from adult insects by using the DNeasy Blood & Tissue Kit (Qiagen, Hilden, Germany). Universal primers (LCO1490: 5′-GGTCAACAAATCATAAAGATATTGG, and HCO2198: 5′-TAAACTTCAGGGTGACCAAAAAATCA) developed by Folmer et al. [12] were used for amplification of the mitochondrial gene of the cytochrome c oxidase subunit I (*COI*). DNA was amplified in a thermal cycler (SureCycler 8800) under the conditions described by Folmer et al. [12]. The amplicons were purified using the Wizard^®^ SV Gel and PCR System Clean-Up kit and sent to Labsergen Langebio (CINVESTAV, Irapuato, Mexico) for sequencing. The resulting sequences were compared in the GenBank nucleotide sequence database [13] using BLAST search software to confirm the genus and to determine percentage identity [14].

### 2.3. Development Time for Immature Stages and Adult Longevity

The developmental time for each immature stage was determined by infesting radish plants. For this, a 5-day-old pair of *D. planipalpis* was placed in a cylindrical cage (473 cm^3^ capacity) that was sealed with a piece of pantyhose fabric held in place with an elastic band. The cylindrical cage contained a radish plant in a small cup (100 mL) with a 1 cm layer of vermiculite in the base. Flies remained inside the cage for 8 h, from 9.00 to 17.00, allowing females to lay eggs. After this period, the number of eggs present on each plant was carefully counted under a Leica EZ4HD stereomicroscope. The number of eggs that hatched on each plant was counted at 24-h intervals for five consecutive days following oviposition. The larvae were left on radish plant tissue for their development. From the twelfth day, the plants were carefully checked every day to separate the pupae. All the pupae collected in a day were placed in plastic containers with moist vermiculite and incubated until adult emergence. The interval between pupal collection and adult emergence was recorded, as was the sex of each insect. Adult emergence was evaluated for three weeks, after which pupae that failed to produce adults were dissected to determine their status. The hatching time for eggs, the development time from egg to pupa and the emergence time for adults (both sexes) were recorded in days. The study was performed on four occasions, each with 8 cages (*n* = 32 cages in total).

Two different experiments were performed to determine the adult longevity of female and male flies in the presence or absence of a host plant. In the first experiment, a recently emerged fly (1–3 h post-emergence) was placed individually in a 473 cm^3^ cage with ad libitum availability of food and water. Longevity was measured for a total of 50 flies of each sex. Mortality was recorded daily until all individuals had died.

In a second experiment, 10 male–female pairs collected just after emergence (1–3 h post-emergence) were placed together in 20 × 20 × 20 cm acrylic cages with continuous access to food and water. A radish seedling was placed in each cage as an oviposition substrate. The test was performed five times with a total of 50 females and 50 males. Mortality was recorded daily until all individuals had died. For both experiments, the median survival time of females and males, with its corresponding 95% confidence interval, was estimated by Kaplan–Meier survival analysis and compared by log-rank test.

### 2.4. Effect of Host Plant on Sexual Maturation

Three different conditions were considered for adult sexual maturation: (i) cages with the stimulus of a radish plant (the main host); (ii) cages with the stimulus of a broccoli plant (var. Gypsy) as an alternative host; and (iii) cages without a host stimulus (no plant as control). A plant of either type, 15–17 cm in height and planted in a 500 mL Styrofoam container with organic substrate, was placed inside an acrylic cage (20 × 20 × 20 cm) covered in nylon mesh on the upper and side walls. Adults of *D. planipalpis* from the laboratory colony were collected at 1–3 h post-emergence and assigned at random to one of the three cage test conditions. Groups of 10–12 adults were separated by sex and placed in each cage. Flies had continuous ad libitum access to hydrolyzed yeast, sugar and water. A subsample of females or males was collected daily from each cage on four consecutive days. This time period was selected based on preliminary tests that indicated that the sexual maturation of both sexes was completed after 4 days. The reproductive system of adults was removed and photographed using a Leica EZ4HD (Leica, Germany) stereomicroscope. Then, the cross-sectional area of the ovaries was measured in females, and the area and length of the testes were measured for males. The average area of both ovaries or testes was calculated for each female or male insect. The head width, considered to be the ocular distance at its widest point across the eyes, was also measured for each insect as a proxy for body size. All measurements were performed using ImageJ software (version 1.4.3.67). The entire procedure was performed on six occasions with different batches of flies and plants, resulting in the dissection of 204 females and 192 males in total.

Gonad measurements were compared by two-way analysis of covariance with plant stimulus and adult age in days as the main factors and with the insect’s head width as a covariate. The normality and homoscedasticity of the data were confirmed by Shapiro–Wilk and Levene’s tests, respectively, prior to analysis. All analyses were performed using the R-based package Jamovi v.2.3.21 [15].

### 2.5. Sexual Behavior

Initially, a brief study was performed to quantify the number of oocytes in the ovaries of mature female flies. For this, groups of 10–12 females (aged 1–3 h post-emergence) were collected from the laboratory colony and were kept together with a similar number of adult males in 20 × 20 × 20 cm acrylic cages until they reached an age of 6–10 days. Sugar, hydrolyzed yeast and water were continuously available. Gravid females of 6–10 days old were dissected under a stereomicroscope and the number of mature oocytes was counted in each of the ovaries for a total of 44 females from six different batches of insects.

To examine sexual behavior, adult flies were separated by sex when aged 2–3 h post-emergence and were marked with a non-toxic acrylic paint dot (Politec^®^, Vinci, Mexico City, Mexico) on the thorax to differentiate all the males and females of the cage. At 9:00 h, on the day after emergence (day 1), seven pairs were placed together in a 25 × 12.5 × 13 cm cage (4 L capacity) that contained a radish plant, 12–15 cm high, with four developed leaves. Adult flies had continuous ad libitum access to food and water. The behavior of the flies was observed continuously from 9:00 to 17:00 over four consecutive days. Several variables were registered during the observation period: mating attempts, mating duration, mating time (hour of the day) and mating site (plant or cage). Female oviposition events were also recorded. Daily, at 17:00, the adults were removed from the cage and separated by sex until the next day, when they were released again in the cage at 9:00. After flies had been removed at 17:00, radish plants were checked, and any eggs were carefully removed and placed on a moist black cloth inside a 90 mm Petri dish. Egg hatching was registered up to five days after each collection. The same plant was used for each of the four consecutive days of the study and then discarded. If a fly died during the experiment, it was replaced by a fly of the same sex and of similar age. Flies were frozen at the end of the experiment. The head width (widest ocular distance) of males was measured as an indicator of body size. The test was performed in seven independent cages with different batches of flies. The frequency of matings registered in the morning (9:00 to 13:00) or afternoon (13:00 to 17:00) was compared by χ^2^ test. To determine whether the size of the male influenced the number of copulations, a generalized linear model (GLM) was fitted with a Poisson distribution and a log-link function.

### 2.6. Effect of Host Plant on Oviposition under Choice and No-Choice Conditions

In the first experiment, oviposition by gravid females on radish was determined. The total number of eggs per day oviposited on a radish plant by a 5-day-old mated gravid female was evaluated over 8 consecutive days. The female was introduced on day 1 and allowed to oviposit for 24 h. After each 24 h period, the female was removed from the cage and placed in a new cage containing a new radish plant. This procedure was performed using 15 individual females for 10 consecutive days, after which time females stopped laying eggs. The number of eggs oviposited on each plant was counted daily under a stereomicroscope. Eggs were directly examined on the plants 3 and 5 days later to evaluate hatching. The mean number of eggs per female per day, mean total eggs laid by each female and mean percentage of hatching eggs were calculated. 

To determine whether virgin females were able to oviposit on radish, a second experiment was performed using unmated females. For this, females were collected from emergence cages (2–3 h post-emergence) and were isolated to avoid mating. When females reached 4 days old, a group of 10 females was introduced into an acrylic cage (20 cm × 20 cm × 20 cm) with a radish plant placed in a 350 mL Styrofoam container with soil. Insects had continuous access to food and water. Radish plants were examined 1, 3, 5 and 10 days later for the presence of eggs. Four replicates were performed, each comprising a group of 10 females.

An additional experiment was performed to evaluate the oviposition of gravid females on radish or broccoli seedlings under choice conditions. Three sets of experiments were performed independently for each of three broccoli varieties, Tlaloc, Emerald and Gypsy, which were each compared to radish as the reference host plant. Experiments were performed in 60 × 60 × 90 cm mesh cages with seedlings 15–20 cm in height. Seedlings were planted in white Styrofoam containers with soil and placed in opposite corners of the cage, the radish plant in one corner and one of the broccoli varieties in the opposite corner, at a distance of approximately 90 cm from one another. A group of five females (5–7 days old) considered to be mated and gravid were released inside the cage at 9:00 and maintained for 24 h with continuous access to food and water. The number of eggs deposited on each seedling was counted after 24 h. The experiment was performed simultaneously in four cages and was repeated twice in each cage, switching the position of the plants on each occasion to avoid a position effect (*n* = 8 replicates). The average number of eggs laid by each female in 24 h was compared between radish and each of the broccoli varieties by paired *t*-test.

Finally, oviposition on three varieties of broccoli was examined under choice and no-choice conditions. Oviposition under choice conditions was evaluated by simultaneously offering *D. planipalpis* flies three varieties of broccoli. The experiment was performed in a 60 × 60 × 90 cm acrylic mesh cage with seedlings of Tlaloc, Emerald and Gypsy varieties that were 15–20 cm in height. Seedlings of the three varieties were planted in white Styrofoam containers (1 plant/container) with soil and placed in an equilateral triangle arrangement at a distance of 50 cm. Five mated females (aged 5–7 days) were released inside the cage at 9:00 and maintained for 24 h with continuous access to food and water. The number of eggs deposited on each broccoli seedling was counted after 24 h. The test was carried out simultaneously in four cages and was performed on three occasions, rotating the position of the seedlings each time (once at each position) (*n* = 9 replicates). The average number of eggs oviposited on plants in 24 h was compared among the three broccoli varieties by fitting a GLM with overdispersion.

To evaluate oviposition under no-choice conditions, five mated females (5–7 days old) were released into a 20 × 20 × 20 cm acrylic cage in which a single broccoli seedling was present in a white Styrofoam container. After 24 h with food and water, the mean number of eggs laid on plants by females was counted. Each of the three broccoli varieties was tested in ten replicate cages. The mean number of eggs oviposited was compared for each variety by analysis of variance (ANOVA), following Shapiro–Wilk and Levene’s tests for data normality and homoscedasticity. Mean separation was achieved by Bonferroni test.

## 3. Results

### 3.1. Taxonomic and Genetic Identification of D. planipalpis

**Figure 1:**
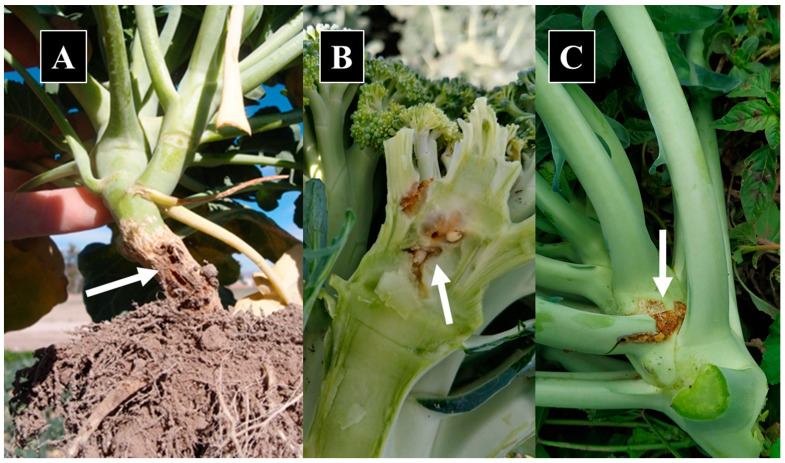
Characteristic damage of *Delia planipalpis* larvae in (**A**) a broccoli plant stem, (**B**) inflorescence and (**C**) leaf axil. Arrows indicate position of larvae.

The taxonomic identification of 50 adults (25 females and 25 males), 20 larvae and 20 pupae collected during the study from the established colony revealed them to be *D. planipalpis*. Two adults, a male and a female, were confirmed by a sequence analysis of the *COI* gene with a 99.69% match in identity to the sequences in GenBank (Table 1). Similarly, twenty-seven pupae collected from damaged broccoli seedlings (Figure 1A) and identified in the adult stage, four larvae collected from damaged leaf axil (Figure 1B) (identified in the adult stage) and five larvae collected from damaged inflorescence (Figure 1C) (identified in the larval stage) matched taxonomically with *D. planipalpis*. Two adults of *D. planipalpis* on the yellow monitoring traps and two larvae collected from the inflorescence were also confirmed as *D. planipalpis* with a 99.69–100% match in sequence identity in the *COI* gene (Table 1). Specimens of *Delia platura* that were also present on the yellow traps were taxonomically identified, and a *COI* sequence analysis indicated a 100% match in identity to that of GenBank sequences (Table 1).

### 3.2. Development Time for Immature Stages and Adult Longevity

The females oviposited a variable number of eggs on the radish plants that fluctuated between 1 and 18 eggs per female per day (average ± SE: 5.3 ± 0.5). A total of 254 eggs were observed among all the replicates. The eggs hatched in an average of 3.3 ± 0.1 days under constant laboratory conditions. From these, 130 pupae (52%) were obtained. Although larval survival was low, not all the deaths can be assumed to be natural, since in some cases the larvae may have been damaged during the inspection process. From the 130 pupae, 82 adults emerged, 43 of which were females (52%) and 39 were males (48%). The average development time of the insects from egg to pupa was highly uniform, and adult emergence from pupae was also highly synchronized and almost identical for both sexes (~14 days) (Table 2). Overall, 37% of the collected pupae did not emerge, even after more than one month. These pupae were dissected and examined and almost all were found to be alive.

**Figure 2:**
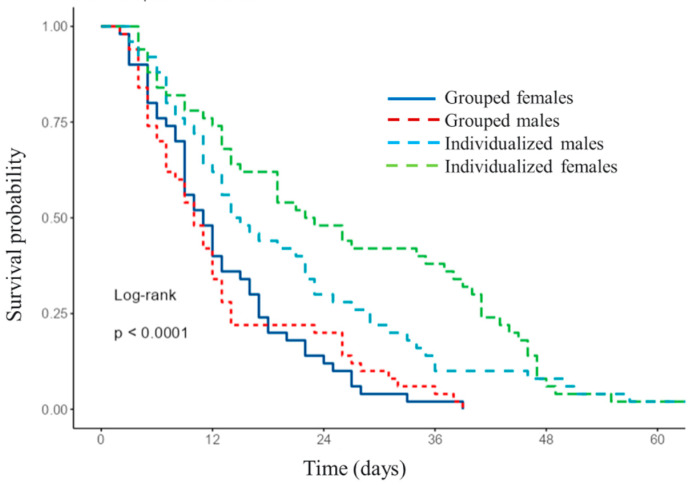
Survival of adult females and males of *Delia planipalpis* when individualized or maintained in groups at 24 ± 1 °C. Both sexes were reared on radish.

The adult survival of radish-reared insects was significantly longer for both sexes when the flies were individualized compared to those maintained in groups (likelihood ratio test χ^2^ = 33.563; df = 3; *p* < 0.001) (Figure 2). The median survival time was similar for the females (11 days; 95% CI = 9–16 days) and males (10 days; 95% CI = 7–13 days) (Kaplan–Meier log-rank, *p* = 0.998) when they were grouped. In contrast, the median survival time was significantly longer for individualized females (22.5 days; 95% CI = 15–39 days) than for individualized males (14.5 days; 95% CI = 12–23 days) (Kaplan–Meier log-rank, *p* = 0.001) (Figure 2).

### 3.3. Effect of Host Plant on Sexual Maturation

**Figure 3:**
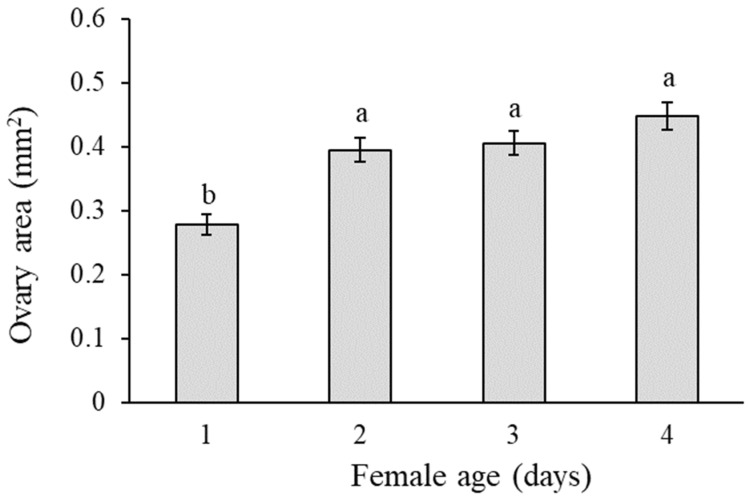
Mean (± SE) cross-sectional area of ovaries of *Delia planipalpis* females 1, 2, 3 and 4 days post-emergence. Different lowercase letters indicate significant differences (ANOVA, Tukey’s test, *p* < 0.05).

For females, the mean cross-sectional area of the ovaries was not significantly influenced by the host plant stimulus (F = 0.320; df = 2, 177; *p* = 0.727), but was significantly lower for females dissected after one day of development compared to females dissected 2, 3 or 4 days post-emergence (F = 15.1; df = 3, 177; *p* < 0.001) (Figure 3). No significant interaction was observed between the plant stimulus and the females’ age (F = 0.527; df = 3, 177; *p* = 0.787). The mean area of the ovaries was significantly correlated with female body size as indicated by the significant head size covariate (F = 137.7; df = 6, 177; *p* <  0.001).

For males, the mean length and width of the testes was not significantly influenced by the host plant stimulus (length: F = 1.609; df = 2, 172; *p* = 0.203; width: F = 1.335; df = 2, 172; *p* = 0.266) or male age (length: F = 0.248; df = 3, 172; *p* = 0.863; width: F = 1.571; df = 3, 172; *p* = 0.198). No significant interaction was observed between the plant stimulus and the males’ age on the testes size (length: F = 0.849; df = 6, 172; *p* = 0.534; width: F = 0.999; df = 6, 172; *p* = 0.428). The size of the testes was significantly affected by body size as indicated by the significant covariate of head width (length: F = 23.445; df = 1, 172; *p* < 0.001; width: F = 3.96; df = 1, 172; *p* = 0.048).

### 3.4. Sexual Behavior

Oocyte production was determined by the dissection of mature females. The average number of oocytes in gravid females varied between 10 and 68 oocytes, with an average (± SE) of 37.4 ± 2.0 oocytes per female.

A total of 51 copulations were recorded. Mating occurred after the male used his forelegs to grip the first abdominal segment of a receptive female, which remained immobile when approached. When a female was not receptive, mainly on the first day post-emergence or after mating, she resisted mating by curving her genitalia under the abdomen and struggled strenuously to escape from the male by vigorously fluttering her wings.

**Figure 4:**
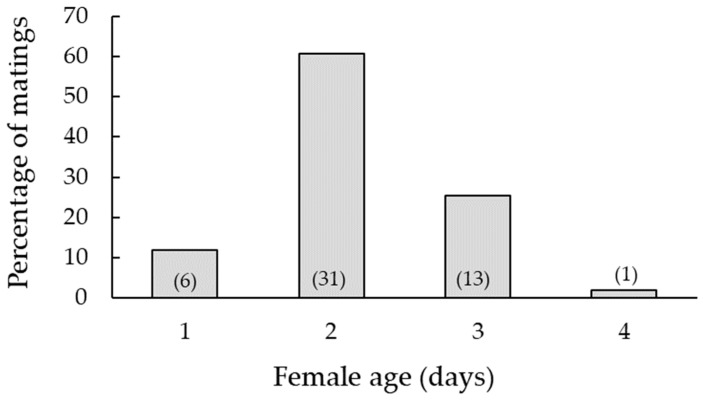
Mating percentage of 1-, 2-, 3- and 4-day-old *Delia planipalpis* after emergence. Numbers within parentheses represent sample size (total *n* = 51).

Almost all the females (95.6%) copulated at some point during the four days of observation (Figure 4). The average copulation time (± SE) was 177 ± 5.8 s. The average age of the females at copulation was 2 days and 7 h. The majority of the matings were recorded on the second and third days following female emergence (Figure 4). Except for four events (5.8%), no re-matings were recorded. Twelve females copulated on the first attempt to mount by a male (25.5% of matings), whereas 26 females were mounted between two and four times before a successful copulation (55.3% of matings). In some cases, females were mounted six to eight times (12.8%) and rarely as many as twelve to sixteen times before a successful copulation (6.4%).

Significantly more copulations were recorded in the morning observation period (9:00–13:00) with 64.7% of the observed matings compared to the afternoon period (13:00–17:00) with 35.3% of the matings (χ^2^ = 8.82; df = 1; *p* = 0.003). The flies copulated at similar frequencies on the plant (51%) or on the cage walls (49%) (χ^2^ = 1.06; df = 1; *p* = 0.302).

Male attempts at copulation were observed immediately after the release of the adults in the cage on the first day. An average of fifteen to sixteen copulation attempts per male was recorded throughout the evaluation period (4 days), equivalent to four attempts per male each day. The size of the male did not influence the number of successful copulations (GLM: χ^2^ = 0.326; df = 1; *p* = 0.568). However, the males continued to attempt mating with apparently non-receptive females, possibly due to the close confinement of both sexes in the laboratory cages.

All the mated females laid fertile eggs, including the females that mated on the first day they were released. Oviposition behavior mainly began on the day following mating. In all cases, the percentage of eggs that hatched in the study was between 93 and 96%.

### 3.5. Effect of Host Plant on Oviposition Behavior under Choice and No-Choice Conditions

Oviposition by gravid and virgin females on radish was quantified in two experiments. The total number of eggs laid by gravid females varied from 9 to 77 eggs per female over 10 days (mean ± SE; 41.2 ± 4.2). The average percentage of eggs that hatched was 91.5 ± 3.7%. In the second experiment, no oviposition by virgin females was observed in any of the four replicates performed.

**Figure 5:**
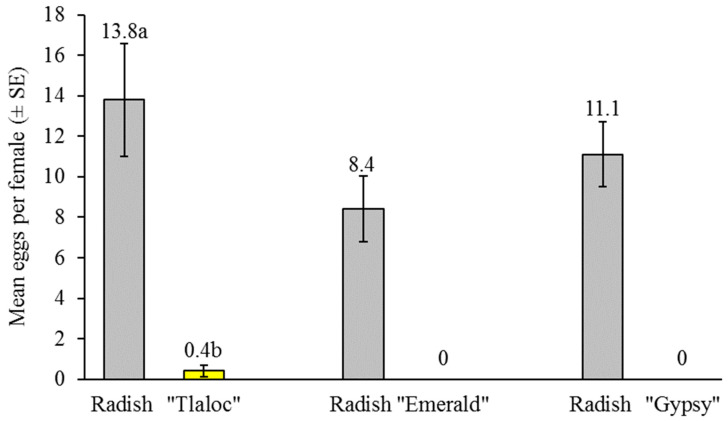
Mean number of eggs oviposited (± SE) per female in choice experiments with radish (gray columns) and three varieties of broccoli (yellow columns) in three separate experiments. Numbers above bars indicate mean values. Numbers followed by different letters differ significantly (paired *t*-test, *p* < 0.05). The results of experiments involving Emerald and Gypsy broccoli varieties were not analyzed statistically due to an absence of oviposition on broccoli.

Oviposition on radish or broccoli was compared under choice conditions. When both radish and broccoli plants were present in cages under choice conditions, a clear preference was observed for oviposition on radish rather than broccoli (Figure 5). This preference was observed for radish over each of the three broccoli varieties tested, i.e., Tlaloc, Emerald and Gypsy. The mean number of eggs per female was markedly higher on radish than on the Tlaloc broccoli (paired *t* = 4.95; df = 7; *p* = 0.002), whereas the results were not analyzed for the Emerald or Gypsy broccoli because there was no oviposition on these varieties (Figure 5). When offered radish plants, the females oviposited mainly on the base of the stem, in the leaf axils and on the root of the radish, although in some cases, eggs were deposited on the new leaf shoots. In contrast, when offered broccoli plants, the females oviposited around the base of the stem or in the adjacent soil within 1 cm from the stem.

**Figure 6:**
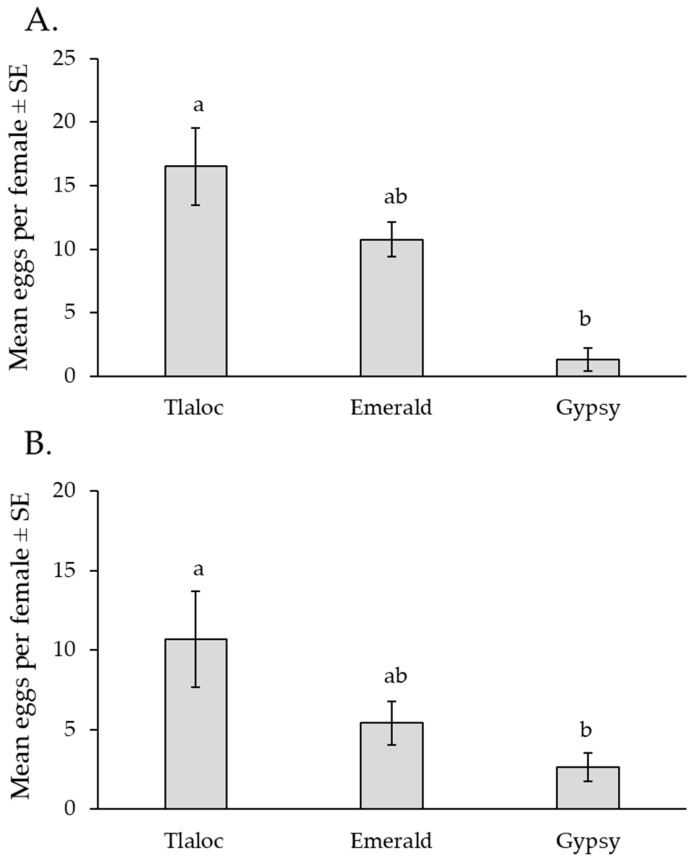
Mean number of eggs (±SE) per female on three varieties of broccoli under choice conditions (**A**) or no-choice conditions (**B**). Columns headed by different letters differ significantly (GLM and ANOVA for A and B, respectively, *p* < 0.05).

Oviposition on the three varieties of broccoli was then compared under choice or no-choice conditions. Under choice conditions, when the three varieties of broccoli were offered simultaneously, the mean number of eggs laid per female was significantly higher on the Tlaloc variety than on the Gypsy variety, while oviposition in the Emerald variety was intermediate (GLM: χ^2^ = 18.9; df = 2; *p* < 0.001) (Figure 6A).

Under no-choice conditions, the mean number of eggs per female also differed significantly among the varieties (F = 4.57; df = 2, 27; *p* = 0.019), with the same pattern as observed in the choice experiment, i.e., the Tlaloc broccoli received significantly higher oviposition than the Gypsy variety, while oviposition on the Emerald variety was intermediate (Figure 6B).

## 4. Discussion

The taxonomic and genetic identification of the adults from the laboratory colony and the specimens collected from damaged broccoli or field traps confirmed the identity of the emerging pest in Mexico as *D. planipalpis*. In fact, *D. planipalpis* was the only species reared from the field-damaged broccoli samples (10 samples with 36 pupae and larvae) collected in 2022, reinforcing the idea that *D. platura* is a secondary species that coexists with *D. planipalpis* but rarely damages the crop [7,16]. The life cycle of *D. planipalpis* from egg to adult lasted approximately 32–33 days at 24 ± 1 °C and was similar to that described by Kelleher [10]. In our study, 30–35% of the pupae did not emerge as adults, possibly due to diapause. Both winter and summer diapause has been reported for other *Delia* species [17,18,19]. Future studies should determine whether *D. planipalpis* enters diapause, for how long and the factors involved in this response.

The maturation of the sexual organs of *D. planipalpis* did not require host plant stimulation, since the ovaries and testes of the adults both developed 24–48 h after emergence, whether or not a radish or broccoli plant was present. Similarly, *D. radicum* and *D. antiqua* matured sexually in the absence of a host plant stimulus [20,21]. Females of *D. planipalpis* reached sexual maturity 1–2 days after emergence, which was similar to that of *D. radicum* at 1–3 days post-emergence [22,23], whereas *D. antiqua* required 6–10 days of maturation post-emergence [21]. Males of *D. planipalpis* were sexually active from the first day after emergence and the females that mated with those males laid fertile eggs. Rapid male maturation was also reported for *D. radicum* males [24]. Studies on other species provide clear evidence that sexual maturation and sexual activity can be altered by the nutritional profile of the host plant [25,26] and by exposure to plant volatile compounds [27], both in Diptera and Lepidoptera [28]. For example, testes size in *Rhagoletis juglandis* (Diptera: Tephritidae) is mainly determined by host plant nutritional characteristics during larval growth [29]. Similarly, ovary maturation and egg load are sensitive to host plant quality in many species of insects (reviewed in Papaj [30]). In addition, exposure to the host plant in the adult stage resulted in an increase in egg load and ovary maturation in the tephritid fly *Anastrepha obliqua* (Diptera: Tephritidae) [31] as well as stimulated testis development and accelerated egg maturation in *Plutella xylostella* (Lepidoptera Plutellidae) [32].

Mating in *D. planipalpis* occurred in a similar manner to that described for *D. radicum*. The *D. planipalpis* females accepted the highest percentage of matings 2–3 days post-emergence, a little earlier than the 4–6 days reported for *D. radicum* [23]. The average duration of copulation was 177 s, very similar to the average of 162 s reported for *D. radicum* [22], but longer than reported for *D. antiqua* (91 s) [33]. When a female was not receptive, she curved her genitalia under the abdomen and struggled vigorously to escape from the male. As has been observed in *D. antiqua* and *D. platura* [34,35], most *D. planipalpis* females mated only once during the 4-day observation period, although it is possible that females may engage in re-mating later in life. Only four re-mating events (<6%) were registered, which may have been influenced by the close proximity of the males held within the cages. The males continued to attempt mating with apparently non-receptive females (>4 attempts per male per day), a behavior also reported for *D. radicum*, in which mating attempts were markedly more frequent, ranging from 5 to 106 attempts per day, which could cause females to mate more than once [22,23]. Most matings in *D. planipalpis* occurred in the morning, a pattern also seen for *D. radicum* [23].

No oviposition was registered for unmated females. A single unmated female was reported to lay eggs in the study by Kelleher [10], but it is clear that unmated oviposition never or rarely occurs in *D. planipalpis*. Similarly, unmated females of *D. antiqua* and *D. radicum* rarely lay eggs [20,22]. Insect oviposition behavior is usually a response to previous mechanical stimulation from the male, the presence of seminal fluid or some specific component of the ejaculate [36,37,38,39]. For example, products of the male accessory glands have been shown to stimulate oviposition in other *Delia* species and other insects [23,40,41,42].

The eggs of *D. planipalpis* were usually well hidden in the soil, often in holes or small cracks at a depth of 5–10 mm. We also observed that a cracked soil structure near the stem of broccoli seedlings could result in increased egg laying by females, as previously reported for *D. radicum* [43], *D. antiqua* [44] and *D. platura* [45]. The number of eggs laid by *D. planipalpis* females on a radish plant during her lifetime averaged 41.2 ± 4.2, a value that closely matched the average number of oocytes per female (37.4 ± 2.0) detected 6–10 days post-emergence and is similar to the value previously reported for this species [10].

We observed a clear preference of *D. planipalpis* from our colony to oviposit on radish rather than on broccoli under choice conditions, but we do not know whether this preference could have been conditioned by previously rearing the flies on radish. On several occasions we attempted to transfer the laboratory colony to broccoli-based rearing, but consistently failed due to the poor oviposition response of the flies to any of the broccoli varieties. Studies on other species of *Delia* indicate that the numbers of eggs oviposited can vary depending on the host plant species, the shape and development of the plant and the soil conditions [43,44,46].

Adult longevity differed between *D. planipalpis* reared individually or in groups. The females’ longevity exceeded that of the males when the insects were individualized, but this difference disappeared when the insects were maintained in groups. Compared to individually isolated insects, there was likely a high energy cost for both sexes when they were present in groups due the sexual activity within groups and the additional ovipositional costs for females. Studies on other species indicate that for male dipterans, these costs arise through courtship, sexual signaling, male–male competition, investment in gametes and seminal fluid, resulting in a decrease in male longevity [47,48,49,50]. As a result, males confined in pairs [51] or with different-sized groups of both sexes experience reduced survival compared with individualized males [52]. In contrast, for female dipterans, costs arise from harassment by males [49] and physical harm during mating [53] or the transfer of toxic seminal fluid [54], as well as the substantial costs of egg production [48,55] that result in reduced female longevity [53,56].

The findings of our study have implications for pest control strategies targeted at this pest. For example, if the apparent preference for radish over broccoli is confirmed in field studies, then radish could be employed as a trap crop at the field margins to lure *D. planipalpis* away from broccoli plants. Moreover, the short interval (typically 1–3 days) between adult female emergence, mating and the start of oviposition means that chemical control measures targeted at adults likely need to be applied at frequent intervals. In contrast, control strategies based on mass trapping are unlikely to provide adequate crop protection as these approaches tend to have a cumulative effect over time. Similarly, the use of fungal bioinsecticides against adults is also unlikely to prove effective due to the slow-acting nature of these products. Finally, the apparently low incidence of re-mating in this pest suggests that it may be a suitable target for the sterile insect technique [57] on an area-wide basis in the broccoli-growing region of Mexico. However, evaluating the feasibility of this technique would require detailed studies on mass-rearing protocols, sterilization procedures and the sexual competitiveness of sterile males, as well as a considerable initial investment in insect rearing and sterilization infrastructure.

## 5. Conclusions

This study has expanded our knowledge of the biology, physiology, sexual behavior and oviposition of *D. planipalpis*. In general, *D. planipalpis* has similar biology and behavior as *D. radicum*, another pest that feeds on crucifers. The females of *D. planipalpis* had a strong preference to oviposit on radish plants, rather than on broccoli plants, although precise conclusions cannot be drawn as our colony was maintained on radish plants. The ability of *D. planipalpis* to discriminate between the different varieties of broccoli was evident, though the physical and chemical cues that elicit oviposition on broccoli or other crucifers remain uncertain and will require future study. Our findings will contribute to the design of future control strategies against this pest in Mexico and possibly in crucifer-growing regions elsewhere.

## Figures and Tables

**Table 1:** Genetic identification of specimens collected from the laboratory colony of *D. planipalpis* or collected from damaged infested broccoli or yellow sticky traps in the field. Two adults of *D. platura* were also sequenced for comparison.

Sample	GenBank Accession Number	Coverage of *COI* Gene (%)	% Identity
*Delia planipalpis * ^1^			
Female, laboratory colony (IEMC.010)	OR121500	98	99.69
Male, laboratory colony (IEMC.011)	OR121501	98	99.53
Female, field trap (IEMC.012)	OR121502	98	99.69
Male, field trap (IEMC.013)	OR121503	98	99.69
Larva, damaged inflorescence in field (IEMC.014)	OR121504	98	100.00
Larva, damaged inflorescence in field (IEMC.015)	OR121505	98	99.69
*Delia platura * ^2^			
Female, field trap (IEMC.016)	OR121506	98	100.00
Male, field trap (IEMC.017)	OR121507	98	100.00

Specimens used for coverage and identity comparison in GenBank: ^1^ *D. planipalpis* BUIC-DIP1675 and ^2^ *D. platura* 07WNP-10869.

**Table 2:** Mean development time in days for different stages of *Delia planipalpis* reared on radish at 24 ± 1 °C.

Stage		Mean Development Time ± SE (Days)
Egg		3.3 ± 0.1 (*n* = 254)
Larval development		15.7 ± 0.1 (*n* = 130)
Pupal development	Females	13.6 ± 0.2 (*n* = 43)
	Males	13.7 ± 0.3 (*n* = 39)

The sample size (*n*) for each stage is indicated in parentheses. Larval development indicates the period between egg hatching and pupation. Pupal development indicates the period between pupation and adult emergence for each sex.

## Data Availability

All the data presented in this study are available upon request from the corresponding authors.
